# Promoting healthy weight in primary school children through physical activity and nutrition education: a pragmatic evaluation of the CHANGE! randomised intervention study

**DOI:** 10.1186/1471-2458-13-626

**Published:** 2013-07-02

**Authors:** Stuart J Fairclough, Allan F Hackett, Ian G Davies, Rebecca Gobbi, Kelly A Mackintosh, Genevieve L Warburton, Gareth Stratton, Esther MF van Sluijs, Lynne M Boddy

**Affiliations:** 1 Physical Activity Exchange, Research Institute for Sport and Exercise Sciences, Liverpool John Moores University, 62, Great Crosshall Street, Liverpool, UK; 2 Faculty of Education, Community, and Leisure, Liverpool John Moores University, IM Marsh Campus, Barkhill Road, Liverpool, UK; 3 Department of Health Sciences, Liverpool Hope University, Hope Park, Taggert Avenue, Liverpool, UK; 4 Research Centre for Sports and Exercise Sciences, College of Engineering, University of Swansea, Swansea, UK; 5 School of Sports Science, Exercise and Health, University of Western Australia, Perth, Australia; 6 MRC Epidemiology Unit & UKCRC Centre for Diet and Activity Research, Institute of Public Health, Cambridge, UK

**Keywords:** Body size, Light physical activity, Accelerometry, Multilevel modelling

## Abstract

**Background:**

This pragmatic evaluation investigated the effectiveness of the Children’s Health, Activity and Nutrition: Get Educated! (CHANGE!) Project, a cluster randomised intervention to promote healthy weight using an educational focus on physical activity and healthy eating.

**Methods:**

Participants (n = 318, aged 10–11 years) from 6 Intervention and 6 Comparison schools took part in the 20 weeks intervention between November 2010 and March/April 2011. This consisted of a teacher-led curriculum, learning resources, and homework tasks. Primary outcome measures were waist circumference, body mass index (BMI), and BMI z-scores. Secondary outcomes were objectively-assessed physical activity and sedentary time, and food intake. Outcomes were assessed at baseline, at post-intervention (20 weeks), and at follow-up (30 weeks). Data were analysed using 2-level multi-level modelling (levels: school, student) and adjusted for baseline values of the outcomes and potential confounders. Differences in intervention effect by subgroup (sex, weight status, socio-economic status) were explored using statistical interaction.

**Results:**

Significant between-group effects were observed for waist circumference at post-intervention (β for intervention effect =−1.63 (95% CI = −2.20, -1.07) cm, p<0.001) and for BMI z-score at follow-up (β=−0.24 (95% CI = −0.48, -0.003), p=0.04). At follow-up there was also a significant intervention effect for light intensity physical activity (β=25.97 (95% CI = 8.04, 43.89) min, p=0.01). Interaction analyses revealed that the intervention was most effective for overweight/obese participants (waist circumference: β=−2.82 (95% CI = −4.06, -1.58) cm, p<0.001), girls (BMI: β=−0.39 (95% CI = −0.81, 0.03) kg/m^2^, p=0.07), and participants with higher family socioeconomic status (breakfast consumption: β=8.82 (95% CI = 6.47, 11.16), p=0.07).

**Conclusions:**

The CHANGE! intervention positively influenced body size outcomes and light physical activity, and most effectively influenced body size outcomes among overweight and obese children and girls. The findings add support for the effectiveness of combined school-based physical activity and nutrition interventions. Additional work is required to test intervention fidelity and the sustained effectiveness of this intervention in the medium and long term.

**Trial registration:**

Current Controlled Trials ISRCTN03863885.

## Background

It is well established that paediatric obesity increases the risk of cardiometabolic disease in later life [1]. Despite evidence to suggest that the prevalence of obesity has plateaued in recent years within the UK [2] and internationally [3], there is no evidence of a decline, and a high proportion of children remain at risk of morbidity. Physical activity (PA), sedentary behaviours, and food intake are key variables implicated in childhood obesity due to their influence on energy balance [4]. Despite this, children on average are insufficiently active [5], engage in excessive sedentary behaviour [6], and have sub-optimal nutritional intake [7,8].

Many intervention projects have been conducted to arrest the increase in child overweight and obesity through single and combined strategies to enhance levels of habitual PA, reduce time spent in sedentary behaviours, and improve nutritional intake. One systematic review of school-based obesity prevention interventions reported that the effects of interventions including both PA and diet behaviours were equivocal with 45% of reviewed studies demonstrating significant intervention effects on body mass index (BMI) [9]. Mixed success in these interventions can be due to the different intervention strategies and variable methodological quality, such as lack of objective measurements of PA [10] and failure to account for relevant confounders in analyses [11]. Despite these weaknesses in the evidence base, it is suggested that school-based interventions that combine PA and diet may help to prevent children becoming overweight in the long term [9]. Furthermore, previous evidence indicates that school-based interventions are more likely to be effective when PA and dietary behaviours are reinforced at home through a family intervention component [9,12].

The school setting is a logical choice as a context for implementing healthy weight interventions due to existing infrastructure, staff, curricula, facilities, policies, and environments that have potential to promote healthy behaviours. In Europe there is limited evidence of successful school curriculum-based interventions focused on PA and/or nutrition, with previous studies reporting improvements in school time PA [13] and vegetable intake [14], but no effects on weight status [15]. Elsewhere, curriculum-based interventions with additional components (e.g., modifications to school meals) have resulted in positive changes in body size outcomes [16,17]. It is postulated that lifestyle interventions to reduce the risk of overweight may be effective if built into school curricula [12], particularly through interdisciplinary curriculum areas such as Personal, Social, and Health Education (PSHE) (in the UK PSHE is distinct from other health-related subjects such as physical education and food technology) [13]. Furthermore, interventions that can be implemented by school personnel in ‘real life’ conditions (i.e., without researcher support and resources) are advocated [15], as these are less costly [13], and are more likely to be integrated within existing curricula and sustained over time.

The Children’s Health, Activity and Nutrition: Get Educated! (CHANGE!) intervention was designed to promote healthy weight in primary school children through a teacher-delivered curriculum-based intervention with family involvement, focused on physical activity and dietary behaviour. The aim of this pragmatic evaluation was to assess the effectiveness of the CHANGE! intervention on measures of body size, PA and food intake.

## Methods

### Participants

The study was conducted in Wigan Borough in north-west England, UK, a large municipality with a population of over 300,000 that is recognised as an area of high deprivation and health inequalities [18]. Eligible schools were identified within pre-defined geographical units known as Neighbourhood Management Areas (NMA). School-level socio-economic status (SES) was defined as the percentage of students per school eligible to receive free school meals. Within each NMA, one high and one low SES school were randomly selected to take part to ensure representation of the diverse geographical and social contexts present within the locale. Twelve primary schools were approached and recruited to the study (100% participation rate). In each school all children within Year 6 (10–11 years old) were invited to take part in the study (N=420). Available resources for this pragmatic evaluation (e.g., staffing, equipment, available time), dictated that 420 was the maximum number of participants that could be recruited to test the feasibility of the intervention, thus statistical methods were not used to determine samples sizes [19]. Written informed parental consent and participant assent were received from 318 children (75.7% participation rate; Comparison n = 152; Intervention n = 166). Approximately 95% of the children were of white British ethnicity, which is representative of the school age population in Wigan [20]. Ethical approval for the study was obtained from the Liverpool John Moores University Research Ethics Committee (application reference # 10/ECL/039).

### Design

**Figure 1:**
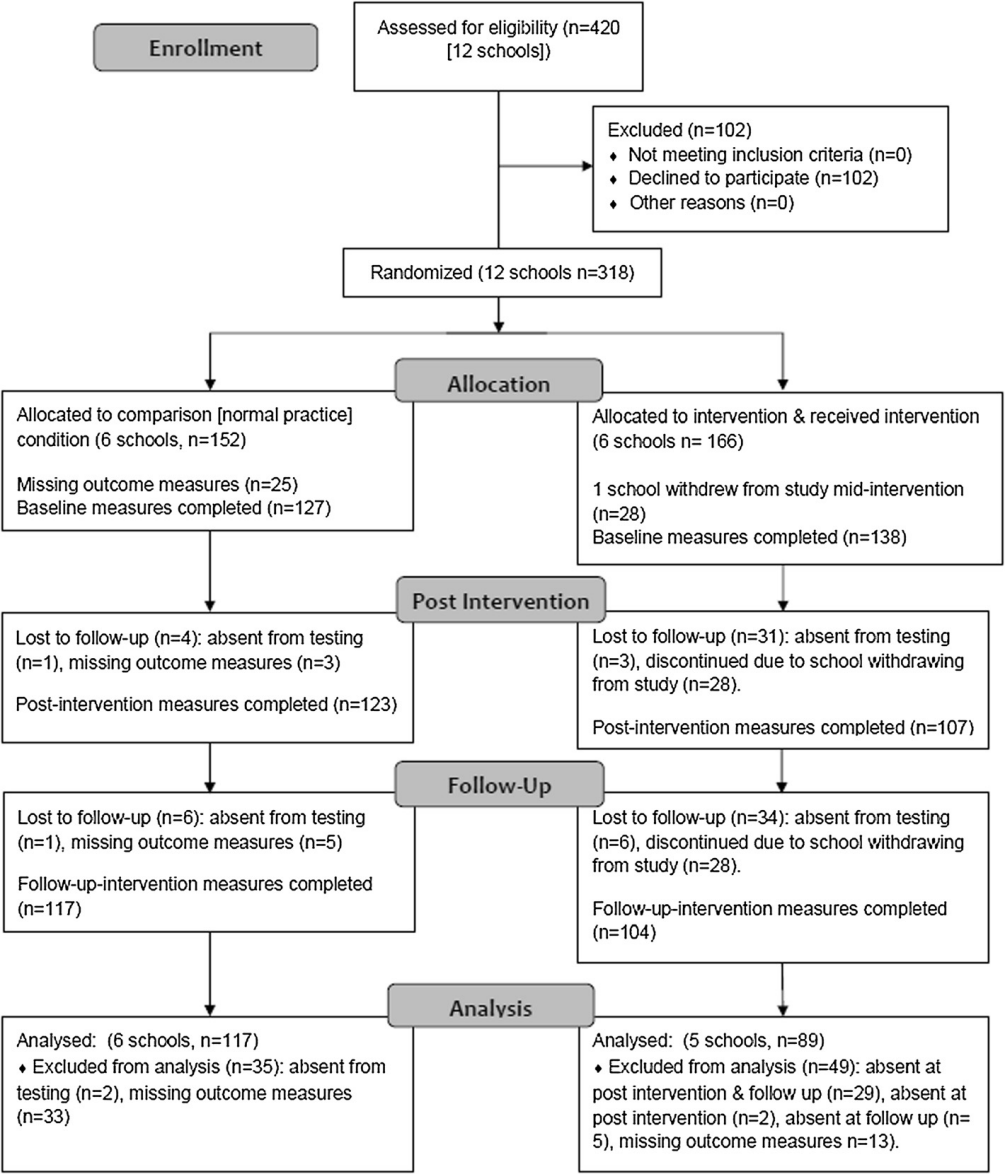
Flow of schools and participants through the study.

Schools were stratified to ensure an equal distribution of high and low SES schools, which were randomly allocated to an Intervention (n=6 schools) or Comparison condition (n=6 schools) using a random number generator (SPSS Inc., Chicago, IL). Due to the nature of the intervention and logistical constraints, randomisation of schools was not blinded and was conducted by the research team prior to baseline measures. Baseline data collection measures were completed in October 2010. Post-intervention measures were completed after the 20 week intervention period in March and April 2011, and follow-up measures were completed 10 weeks after post-intervention measures, prior to school summer holidays. One Intervention school withdrew from the study due to reasons external to the project, prohibiting collection of follow-up data at this school. Full details of the flow of schools and participants through the study are provided in Figure 1.

### Intervention

The CHANGE! Project is a school-based PA and healthy eating intervention study delivered through the PSHE strand of the primary school curriculum. The project was underpinned by social cognitive theory which focuses on the interaction between social and environmental factors on behaviour [21]. The intervention design and content were informed by formative work conducted with parents, children, and teachers in 10 of the schools in the year prior to intervention commencement [22,23]. The final CHANGE! curriculum was adapted from existing resources that have been successfully implemented in the USA [24] and UK [25,26], and which were designed for interdisciplinary curricula [24]. The PSHE curriculum in English primary schools is structured in an interdisciplinary manner with relevant topics delivered collectively within particular themes (e.g., PA and nutrition topics taught within a ‘healthy lifestyles’ theme). With the permission of the publishers of the existing resources, modifications were made to the language, guidelines for diet and physical activity, and reference to local contexts. Year 6 class teachers from the intervention schools received 4 hours of training in the delivery of the curriculum resource, and so were fully familiarised with the curriculum prior to implementation. The CHANGE! curriculum consisted of 20 weekly lesson plans (see Table 1), worksheets, homework tasks, lesson resources, and a CD-ROM. The lessons were of 60 minutes duration and provided an opportunity for children to discuss, explore, and understand the meaning and practicalities of PA and nutrition as key elements of healthy lifestyles. The core message of the PA and sedentary behaviour components was “move more, sit less” with no specific prescription given as to what forms of PA the children should do. The nutrition components focused on topics such as, energy balance, macronutrients, and eating behaviours. The homework tasks supplemented the classroom work and targeted family involvement in food and PA related tasks [27]. The CHANGE! topics were aligned with the UK Healthy Schools programme and were cross-referenced to the England National Curriculum objectives in Physical Education, Science, Maths, English, ICT, History, Geography, as well as PSHE [28]. Classes in the Comparison schools received normal instruction. This did not involve a specific unit of PSHE focused on healthy eating and PA, but concepts related to these areas may have been touched on informally during other lessons (e.g., science, food technology, physical education, etc.).

**Table 1:** CHANGE! Themes, lesson titles and content summary

**Theme**	**Lesson titles**	**Content summary**
Introduction	Healthy Living	Lifestyle options, choices and consequences; eatwell plate
Introduction: What is PA and where do we do it?	Map maker	PA definitions, intensities, guidelines for health, opportunities in local environment [mapping], types of activities
Monitoring and goal setting	Go for goal	Simple monitoring of PA [diary/pedometer], goal setting principles
Reducing sedentary time	Power down	
Impact of technology	Identifying sedentary behaviours, when they occur, how technology has changed our lifestyles, goal setting for reducing screen time	
Components of fitness	Muscle mysteries	
The human heart	Simplify the concept of fitness as representing ‘heart health’, ‘muscle health’, ‘body composition’; incorporate FITT principle as means of enhancing fitness, basic physiological principles to demonstrate effects of PA on body [e.g., pulse rate, etc.]	
Energy balance	Keeping the balance	Fuel; intake; expenditure; balance; negative/ positive; monitoring; nutrient functions and sources
Carbohydrate	Carb smart	Types; processing; starchy foods; why important; fibre; good sources
Sugar	Sugar water	
Beverage buzz	Terminology & types; requirement; labels; sources - hidden; amounts; added sugar; consumption calculations	
Fat	Hunting hidden fat	Terminology & types; requirement; labels (graphing activity); sources; effect of cooking; fish oils
Fruit & vegetables	Menu monitoring	Benefits (source of variety of nutrients); portions; preparation; variety, storage; cooking; access; other foods containing fruit & vegetables, menu planning
Breakfast	Brilliant breakfast	Benefits (energy); portions; choices; sugar; salt; nutritional comparison of different types of breakfast
Snacks (fat/sugar/salt)	Snack attack	
Snack decisions	Frequency of eating; swaps; snacks at bedtime; requirements; hidden sources of fat/sugar/salt; amounts	
Variety	Balancing act	
Keeping the balance	Why variety needed; balanced diet & eatwell plate; nutrient functions and sources; food swaps; access; monitoring task	
Awareness	Foods around the world	Food production – growing; local specialities; history; access; food miles; mapping locality.
Summary	Have you CHANGE!’d?	Summary of principles of healthy living

### Outcome measures

#### Primary outcomes: body size

Stature and sitting stature to the nearest 0.1 cm (Seca Ltd. Birmingham, UK) and body mass to the nearest 0.1 kg (Seca Ltd. Birmingham, UK) were measured using standard techniques [29]. Body mass index was calculated (body mass (kg) / stature^2^ (m^2^)) and BMI z-scores were assigned to each participant [30]. Waist circumference was measured to the nearest 0.1 cm using a non-elastic anthropometric tape and measurements were taken at the narrowest point between the bottom of the ribs and the iliac crest. All measurements were undertaken by the same trained researchers.

#### Secondary outcomes

##### Physical activity and sedentary time

Physical activity was objectively assessed for 7 consecutive days using ActiGraph GT1M accelerometers (ActiGraph LLC, Pensacola, FL). The ActiGraph is a common tool used to assess the volume and intensity of PA, and it has previously been validated with children [31]. To distinguish between wear time and sleep time children also completed a log sheet to record when the ActiGraph was put on in the morning and removed at night before bed, and any other times when the monitor was removed (e.g., during showering, contact sports, swimming etc.). These log sheets were checked and initialled by parents at the end of each day. During the monitoring period physical activity was recorded using 5 second epochs [32]. Sustained 20 minute periods of zero counts were considered non-wear time [33]. Children were included in the data analysis if they wore the monitors for at least 540 minutes on week days [34] and 480 minutes on weekend days [35] for a minimum of 3 days in total [36]. These inclusion criteria have previously shown acceptable reliability in similarly aged children [36]. Numbers of participants that did not meet these criteria were 38 (11.9%) at baseline, 60 (20.6%) at post-intervention, and 77 (26.6%) at follow-up. There were no significant differences in descriptive characteristics between included and excluded children at baseline (p=0.08-0.76), post-intervention (p=0.12-0.96), or follow-up (p=0.50-0.98). Furthermore, no differences in ActiGraph compliance were observed between Intervention and Comparison groups. As there is no consensus as to which ActiGraph cutpoints are the most appropriate in diverse paediatric populations, a sub-study was conducted which developed a field-based protocol for generating population-specific accelerometer cut points. Cut points of >100 and <2160 counts per min, ≥2160 counts per min, and ≥4806 counts per min classified light intensity physical activity (LPA), moderate intensity physical activity (MPA), and vigorous intensity physical activity (VPA), respectively [37]. For sedentary time a cut point of 100 counts per minute was used [38].

#### Food intake

Participants completed a 24 hour recall food intake questionnaire [39]. The survey has acceptable validity [40], reliability [41], and has been widely used in similarly aged children [42,43]. The survey contains 62 food items included some of the most commonly consumed among this age group, such as breakfast cereals, breads, meats and dairy products. In addition, children reported whether they had eaten breakfast, fruit, and vegetables on the day prior to survey completion. These three items were considered as being consistent with a ‘healthy diet’ based on existing research evidence [44] and public health messages (e.g., ‘5-a-day’) and were therefore used as the food intake outcomes in the analyses.

### Assessment of covariates

International Obesity Task Force age and sex-specific body mass index (BMI) cut-points [45] were used to classify children as either normal-weight (NW) or overweight/obese (OW). Somatic maturity status was estimated by maturity offset values (i.e., years from attainment of peak height velocity [APHV]), which were calculated using sex-specific regression equations that included stature, sitting stature, leg length, chronological age, body mass (girls only), and their interactions [46]. The 20 m shuttle run test (20 m SRT) was conducted to provide an estimate of cardiorespiratory fitness (CRF). This test has been widely used in children of similar age [47-49].

Family SES was defined using home post code to generate indices of multiple deprivation (IMD) scores. IMD scores are a composite of seven domains of deprivation (income, employment, education, health, crime, access to services, and living environment) [50] with higher scores representing higher degrees of deprivation. IMD scores were ranked and the median calculated. Participants in the upper and lower 50th percentiles represented low and high SES groups, respectively. The number of children enrolled in each school was recorded. An estimate of playground spatial area was calculated using aerial views of the schools’ playground areas, located using the Google™ Earth Pro (GEP) application (version 6.1.0.4738). Playground areas were calculated using the GEP polygon tool and summed for each school [51]. Daily temperature and rainfall were recorded [52] using local weather centre data.

### Statistical analysis

Multilevel linear and logistic regression analyses examined continuous and dichotomous outcome measures, respectively. Multilevel models can analyse the hierarchical nature of non-independent, nested data by taking into account the dependency of observations [53]. The outcome measures at two follow-up measurements (i.e., 20 weeks post-intervention and 30 weeks follow-up) were the dependent variables. To account for the students being nested in schools, a 2-level data structure was used. Children were defined as the first level unit of analysis, and school was the second level unit of analysis. Separate analyses were conducted to assess intervention effects between baseline and post-intervention, and baseline and follow-up. Preliminary analyses inspected between-group differences in baseline values of potential confounding variables. Where statistically significant between-group differences existed, these variables were included in the adjusted multilevel models as covariates. Regression coefficients for the group variables (where ‘0’ indicated Comparison schools and ‘1’ indicated Intervention schools) reflected between-group differences in the outcome measures (adjusted for baseline values and covariates). Potential effect modification was assessed using interaction terms with dichotomous covariates (i.e., sex, weight status group, and SES group) to investigate whether intervention effects differed between subgroups [54]. Separate ’crude’ interaction analyses adjusted for each interaction term, group, and baseline value of the outcome measure were initially performed. Where these were significant, ‘adjusted’ interaction analyses (i.e., interaction term included in adjusted multilevel models) were conducted for each effect modifier [54]. Regression coefficients in the main and interaction models were assessed for significance using the Wald statistic. Analyses were performed using MLwiN 2.26 software (Centre for Multilevel Modelling, University of Bristol, UK). Statistical significance was set at p<0.05, and at p<0.1 for interaction terms [54].

## Results

### Preliminary results

Participant retention ranged from 84% (baseline) to 77% (follow-up) in the Comparison Group. The Intervention group’s retention ranged from 83% at baseline to 63% at follow-up. However, the withdrawal of one school mid-way through the intervention automatically excluded 28 children. Had the school not withdrawn and assuming all the children continued through the study, the retention at follow-up would have been 80%. Baseline variables did not differ between children who completed the study and those that were excluded from the analyses (p>0.05). Characteristics of the participants at baseline are presented in Table 2. Comparison children recorded significantly better 20m SRT performances (p=0.003) and Intervention children had significantly higher IMD scores (p=0.007). The number of enrolled children and playground area per child were significantly greater in Comparison schools (p<0.001). During physical activity data collection, average temperature was lower and rainfall greater in Comparison schools (p<0.001).

**Table 2:** Baseline descriptive characteristics, body size, physical activity levels, sedentary time, 20 m SRT performance, and food intake of comparison and intervention children (Mean ± SD except weight status and food intake)

	**Group**					
**Measure**	**Comparison**			**Intervention**		
	**Boy**	**Girl**	**All**	**Boy**	**Girl**	
Age (yrs)	10.7 (0.3)	10.7 (0.3)	10.7 (0.3)	10.6 (0.3)	10.6 (0.3)	10.6 (0.3)
Stature (m)	1.4 (0.1)	1.5 (0.1)	1.4 (0.1)	1.4 (0.1)	1.4 (0.1)	1.4 (0.1)
Body mass (kg)	35.3 (7.9)	39.9 (11.1)	38.1 (10.2)	35.7 (6.3)	36.1 (9.0)	36.2 (7.9)
Maturity offset (yrs from APHV)	−3.1 (0.4)	−1.3 (0.6)	−2.0 (1.1)	−3.0 (0.4)	−1.4 (0.6)	−2.1 (1.0)
SES (IMD score)	26.0 (15.0)	23.1 (13.8)	24.2 (14.3)	27.2 (18.0)	30.7 (18.9)	29.2 (18.5)
BMI (kg/m^2^)	17.0 (2.9)	18.8 (4.0)	18.1 (3.7)	17.5 (2.4)	18.1 (3.4)	17.9 (3.0)
Weight status:						
Normal weight (%)	82.6	74.7	78.3	88.6	71.8	79.9
Overweight/obese (%)	17.4	25.3	21.7	11.4	28.2	20.1
BMI-z-score	−0.2 (1.4)	0.3 (1.4)	0.1 (1.4)	0.2 (1.0)	0.1 (1.3)	0.2 (1.1)
Waist circumference (cm)	60.7 (7.9)	63.1 (9.1)	62.2 (8.7)	61.6 (6.1)	61.3 (8.7)	61.5 (7.6)
Physical activity:						
LPA (min/day)	182.9 (31.5)	176.8 (23.9)	179.3 (27.26)	167.5 (27.7)	175.8 (30.2)	172.2 (29.3)
MPA (min/day)	49.1 (11.4)	37.8 (9.9)	42.3 (11.8)	55.1 (14.1)	43.8 (12.4)	48.9 (14.3)
VPA (min/day)	17.5 (9.0)	11.1 (5.0)	13.6 (7.5)	18.8 (8.5)	14.1 (7.1)	16.2 (8.1)
Sedentary time (min/day)	505.2 (68.6)	519.8 (58.5)	513.9 (62.9)	505.2 (55.0)	498.1 (57.0)	501.2 (56.0)
20 m SRT performance (shuttle runs)	39.2 (19.5)	25.8 (13.0)	31.1 (17.1)	30.9 (14.4)	20.8 (10.0)	25.4 (13.1)
Food consumption:						
Breakfast (%)	89.7	91.6	90.7	92.4	89.4	90.9
Fruit (%)	73.1	71.3	72.1	66.7	77.1	72.0
Vegetables (%)	60.3	48.1	53.7	49.4	48.2	48.8

### Intervention effects

**Figure 2:**
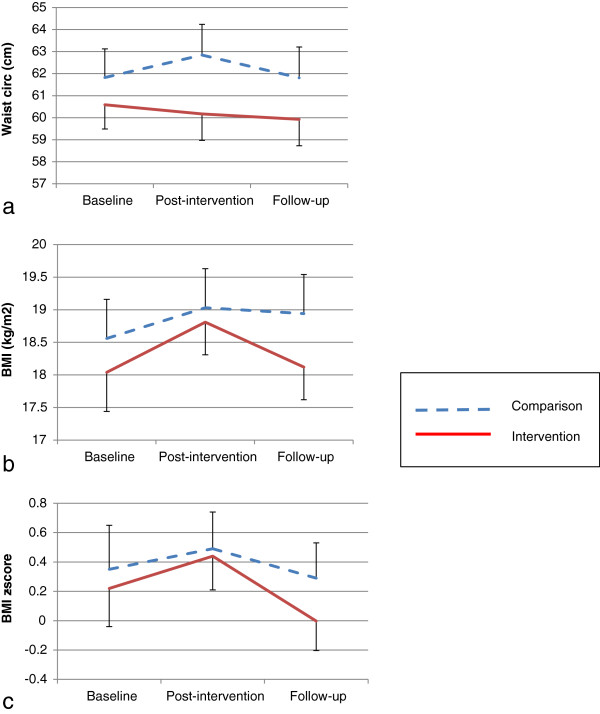
**Adjusted means (SE) of body size outcomes across each time point. a**: waist circumference; **b**: BMI; **c**: BMI z-score.

In adjusted analyses significant between-group intervention effects were observed between baseline and post-intervention for waist circumference (β=−1.63 (95% CI = −2.20, -1.07) cm, p<0.001). No other significant intervention effects were observed in these analyses (Table 3). Between baseline and follow-up (Table 4) there were significant effects for BMI z-score (β=−0.24, (95% CI = −0.48, -0.003), p=0.04) and LPA (β=25.97 (95% CI = 8.04, 43.89) min, p=0.01). At follow-up non-significant between group differences were observed for BMI (β=−0.47 (95% CI = −1.03, 0.09)) and sedentary time (β=−8.44 (95% CI = −53.23, 36.35) min). Adjusted means of the body size outcome measures across each time point are presented in Figure 2. No significant intervention effects were observed for MPA, VPA, and previous day breakfast, fruit and vegetable intake.

**Table 3:** Multilevel analyses of the effectiveness of the CHANGE! intervention between baseline and post-intervention

	**Crude model**^**a**^			**Adjusted model**^**b**^		
**Outcome measure**	**β or OR (95% CI)**	**ICC**	**P**	**β or OR (95% CI)**	**ICC**	**P**
Body size:						
Waist circumference (cm)	**−1.83**^**c **^**(−2.38, -1.29)**	0.004	**<0.001**	**−1.63**^**c **^**(−2.20, -1.07)**	**0.00**	**<0.001**
BMI (kg/m^2^)	0.06^c^ (−0.30, 0.42)	0.05	0.74	0.10^c^ (−0.37, 0.38)	0.05	0.98
BMI z-score	−0.01^c^ (−0.19, 0.17)	0.04	0.92	−0.04^c^ (−0.22, 0.15)	0.03	0.68
Physical activity:						
LPA (min/day)	−6.21^c^ (−16.29, 3.86)	0.08	0.23	5.14^c^ (−10.29, 20.57)	0.00	0.51
MPA (min/day)	−1.23^c^ (−6.08, 3.62	0.10	0.62	1.67^c^ (−7.35, 10.68)	0.04	0.72
VPA (min/day)	−0.22^c^ (−2.64, 2.20)	0.05	0.86	2.85^c^ (−1.64, 7.35)	0.00	0.21
Sedentary time (min/day)	0.51^c^ (−18.84, 19.87)	0.01	0.96	28.35^c^ (−14.88, 71.58)	0.00	0.20
Food:						
Breakfast	1.21^d^ (0.38, 2.04)	-	0.65	1.13^d^ (0.02, 2.22)	-	0.84
Fruit	0.75^d^ (0.14, 1.36)	-	0.35	0.81^d^ (0.42, 1.20)	-	0.60
Vegetables	1.22^d^ (0.65, 1.79)	-	0.33	1.17^d^ (1.02, 1.32)	-	0.64

Notes. *OR* odds ratio, *CI* confidence interval, *ICC* intra-class correlation. Values reflect the intervention effects (i.e., between-group differences) between baseline and post-intervention. Values in bold denote beta (95% CI) and significance values of outcomes with significant intervention effects (P<0.05).

^a^Adjusted for group and baseline value of the outcome measure.

^b^Additionally adjusted for, sex, SES group, weight status group (physical activity outcome measures were also adjusted for playground area, 20 m SRT performance, average weekly temperature and rainfall at baseline; food outcome measures were also adjusted for MVPA at baseline).

^c^β value.

^d^Odds ratio.

**Table 4:** Multilevel analyses of the effectiveness of the CHANGE! intervention between baseline and follow-up

	**Crude model**^**a**^			**Adjusted model**^**b**^		
**Outcome measure**	**β or OR (95% CI)**	**ICC**	**P**	**β or OR (95% CI)**	**ICC**	**P**
Body size:						
Waist circumference (cm)	**−0.95**^**c **^**(0.98, -0.92)**	0.10	**0.06**	−0.64^c^ (−1.50, 0.22)	0.06	0.15
BMI (kg/m^2^)	−0.55^c^ (−1.16, 0.06)	0.22	0.76	−0.47^c^ (−1.03, 0.09)	0.18	0.09
BMI z-score	**−0.27**^**c **^**(−0.53, -0.01)**	0.21	**0.04**	**−0.24**^**c **^**(−0.48, -0.003)**	0.17	**0.04**
Physical activity:						
LPA (min day)	−2.40^c^ (−18.05, 13.25)	0.19	0.76	**25.97 (8.04, 43.89)**	0.04	**0.01**
MPA (min/day)	−3.92^c^ (−9.94, 2.10)	0.07	0.20	−3.27^c^ (−13.02, 6.48)	0.00	0.52
VPA (min/day)	−1.12^c^ (−3.16, 0.92)	0.00	0.28	1.73^c^ (−3.25, 6.71)	0.00	0.50
Sedentary time (min/day)	−0.02^c^ (−18.79, 18.76)	0.00	0.99	−8.44^c^ (−53.23, 36.35)	0.00	0.97
Food:						
Breakfast	0.85^d^ (0.03, 1.67)	-	0.70	1.88^d^ (0.76, 2.99)	-	0.27
Fruit	1.35^d^ (0.69, 2.01)	-	0.37	0.99^d^ (0.24, 1.74)	-	0.99
Vegetables	1.07^d^ (0.39, 1.75)	-	0.62	1.06^d^ (3.45, 1.78)	-	0.87

Notes. *OR* odds ratio, *CI* confidence interval, *ICC* intra-class correlation. Values reflect the intervention effects (i.e., between-group differences) between baseline and follow-up. Values in bold denote beta (95% CI) and significance values of outcomes with significant intervention effects (P<0.05).

^a^Adjusted for group and baseline value of the outcome measure.

^b^Additionally adjusted for, sex, SES group, weight status group (physical activity outcome measures were also adjusted for playground area, 20 m SRT performance, average weekly temperature and rainfall at baseline; food outcome measures were also adjusted for MVPA at baseline).

^c^β value.

^d^Odds ratio.

### Interaction effects

Table 5 shows the results of the significant sub-group interaction effects. The post-intervention interaction effect of the CHANGE! curriculum on waist circumference was stronger in OW participants (β =−2.82 (95% CI = −4.06, -1.58) cm, p<0.001) than in NW participants (β =−1.34 (95% CI = −2.00, -0.72) cm, p<0.001). At post-intervention BMI (β =−0.39 (95% CI = −0.81, 0.03) kg/m^2^, p=0.07) and BMI z-score (β =−0.18 (95% CI = −0.42, 0.06) cm, p=0.14) were strongest in girls whereas both outcomes increased in boys. The post-intervention effect on breakfast consumption was strongest in the high SES group (OR=8.82 (95% CI = 6.47, 11.16), p=0.07). There were no significant interactions with PA or sedentary time.

**Table 5:** Significant post-intervention Intervention sub-group interactions

	**Body size**						**Physical activity**		**Food intake**	
	**Waist circumference**		**BMI**		**BMI z-score**		**LPA**		**Breakfast**	
**Interactions**	**Β (95% CI)**	**p**	**Β (95% CI)**	**p**	**Β (95% CI)**	**p**	**Β (95% CI)**	**p**	**OR (95% CI)**	**p**
Intervention * sex (crude)	0.46 (−0.57, 1.49)	0.38	**0.77 (0.31, 1.23)**	**0.001**	**0.34 (0.08, 0.59)**	**0.01**	**12.84 (0.18, 25.50)**	**0.04**	1.18 (−0.50, 2.86)	0.76
Girls	n/a	-	**−0.39 (−0.81, 0.03)**	**0.07**	−0.18 (−0.42, 0.06)	0.14	−1.05 (−18.10, 16.0)	0.92	n/a	-
Boys	n/a	-	**0.47 (0.03 ,0.91)**	**0.04**	**0.27 (0.02, 0.52)**	**0.04**	10.25 (−6.31, 26.81)	0.22	n/a	-
Intervention x weight status (crude)	**−1.38 (−2.70, -0.05)**	**0.04**	0.10 (−0.51, 0.71)	0.75	−0.01 (−0.33, 0.32)	0.97	1.10 (−14.58, 16.78)	0.89	**10.30 (9.30, 11.30)**	**0.06**
NW	**−1.34 (−2.00, -0.72)**	**<0.001**	n/a	-	n/a	-	n/a	-	0.67 (0.59, 1.94)	0.53
OWOB	**−2.82 (−4.06, -1.58)**	**<0.001**	n/a	-	n/a	-	n/a	-	5.08 (2.73, 7.43)	0.17
Intervention * SES (crude)	0.44 (−0.65, 1.53)	0.42	−0.11 (−0.64, 0.42)	0.68	0.01 (−0.28, 0.30)	0.95	−1.49 (−15.07, 12.09)	0.82	**7.69 (5.66, 9.72)**	**0.05**
High SES	n/a	-	n/a	-	n/a	-	n/a	-	**8.82 (6.47, 11.16)**	**0.07**
Low SES	n/a	-	n/a	-	n/a	-	n/a	-	0.38 (−1.04, 1.80)	0.19

Notes. Crude analyses (adjusted for interaction terms, group, baseline value of the outcome measure) of interaction terms to evaluate potential effect modification are shown. Where these were significant, adjusted analyses (i.e., interaction term included in adjusted multilevel models) were conducted with results for each effect modifier shown (e.g., for sex, girls’ and boys’ results are reported). Values in bold denote beta (95% CI) and significance values of outcomes with significant intervention effects (P<0.10).

## Discussion

The CHANGE! intervention was effective in promoting healthy weight through educational activities focused on increased PA, healthy eating, and reduced sedentary time. Positive intervention effects were observed for body size outcomes, with significant between-group differences identified for waist circumference at post-intervention (−1.63 cm), and BMI z-score at follow-up (−0.24). Waist circumference [55] and BMI z-scores [56] are positively associated with cardiovascular disease risk in children and the changes in waist circumference and BMI z-scores of the magnitudes observed here have previously been reported as sufficient for population health benefit [57]. Thus, the positive effects of the CHANGE! intervention on body size outcomes were likely to be of clinical benefit at the population level, and are consistent with previous school-based interventions focused on physical activity and diet. The Lekker Fit! study reported a 0.71 cm decrease in waist circumference among 9–12 year old Intervention children compared to Comparison group peers [58]. Moreover, significant decreases in intervention children’s BMI z-scores (0.2) were observed after two years follow-up in the APPLE Project [59], and in the Planet Health study obesity prevalence was significantly reduced in girls [24]. However, other combined physical activity and diet focused interventions have been less effective in reducing measures of body size and obesity prevalence [13,60-62]. Insufficient statistical power to detect changes [13], measurement error [63,64], and lack of group-specific intervention content [24,65] are cited as possible reasons for lack of intervention effects in these studies. Lack of statistical power is relevant to some of the analyses in our study, where potentially meaningful yet non-significant effects on waist circumference and BMI were noted at follow-up.

There was a significant between group difference in LPA at follow-up. The CHANGE! lessons used a generic approach to promoting increased physical activity and reduced sedentary behaviour which focused on a simple message of “move more, sit less”. In this sense the Intervention children were not directed to participate in specific physical activity modes or intensities. We felt that this non-prescriptive approach would be more ecologically valid as the focus was on habitual physical activity and sedentary time. The between group differences in LPA of 5.1 minutes at post-intervention and 26 minutes at follow-up suggest that the Intervention children engaged in more incidental physical activity, even though this was below the moderate intensity threshold commonly acknowledged as beneficial for health [66]. Recent evidence though suggests that LPA may also play a role in health promotion. For example among 11-year old boys and girls objectively assessed LPA was negatively associated with DEXA derived fat mass [67]. Similar findings have been reported by others [68-70] but these may be moderated to an extent by sex [69]. The evidence supporting inverse relationships between LPA and body size related outcomes is however equivocal with other authors reporting no associations [71,72]. Nevertheless, the role of LPA in health risk reduction may be growing more prominent [73]. Recent commentary on this topic in adults highlights that reductions in mortality risk begin with increases in activity beyond baseline (i.e., no activity or sedentary), and that the rate of risk reduction is greatest among the least active members of the population [74]. The contention is that LPA is beneficial to health when sedentary behaviours are replaced by LPA, and MPA and VPA are constant [74], and therefore total energy expenditure is increased [66]. Though this relationship between LPA and sedentary time was not observed at post-intervention, the significant effects for LPA at follow-up did coincide with a between-group difference of −8.4 minutes sedentary time. As MPA and VPA were relatively unchanged the positive effects on LPA and sedentary time support the notion that LPA is of value in the context of total energy expenditure. Furthermore, LPA may be more important for the least active children, such as girls and the OW group. It is perhaps significant that sub-sample analyses demonstrated greatest effects in these groups in relation to BMI (girls) and waist circumference (OW).

No intervention effects were observed for MPA and VPA. It is likely that relying solely on a curricular intervention to illicit significant change in these relatively higher PA intensities was insufficient. In the school setting, environmental and/or policy intervention components would most likely have complemented the curricular and homework elements of CHANGE! to increase MPA and VPA [11,75]. For example, in Australia the Fit-4-Fun intervention which included modifications to the recess environment and prescribed family engagement activities to complement the curriculum intervention component, reported improvements in BMI, BMI z-score, and PA [76]. In CHANGE!, however, resources were not available and policy changes were not forthcoming to modify the likes of the recess environment (e.g., playground markings, equipment availability), physical education class content and delivery, access to school facilities out of hours, etc. Although the Intervention children recorded less sedentary time than the Comparison group at follow up, at post-intervention they did over 28 minutes more. It is possible that the children did not act upon the intervention messages regarding sedentary behaviours, or that the messages were not sufficiently emphasised either in the lesson plans or in the lesson delivery. While plausible, this explanation is limited though by the absence of lesson observations or teacher evaluations.

No intervention effects were observed for the selected day food intake outcomes. The relatively short duration of the CHANGE! intervention and the dichotomous response structure of the previous day food intake measure offer some explanation why this was the case. Moreover, high baseline values observed for these outcomes suggests a ceiling effect may have been evident whereby it was not possible to detect children with significantly better or worse food intakes than others. This phenomenon is not uncommon when assessing behavioural outcomes in school-based interventions [13].

Sub-group analyses highlighted how intervention effects for BMI were significantly greater in girls than in boys. These findings endorse the contention that gender is a significant moderator of school-based energy balance behaviour interventions, which appear to typically work better for girls than boys [54]. Indeed, the significant post-intervention increases in the intervention boys’ BMI and BMI z-score values reinforce this viewpoint. A significant intervention effect on waist circumference was evident for all Intervention children, but was stronger in OW children compared to NW children at post-intervention. This demonstrates that not only was the CHANGE! intervention effective for children across the weight status spectrum, but that it was particularly effective for those who were initially overweight or obese, and who therefore were at greatest potential risks of poor health. In developed countries prevalence of overweight and obesity is highest in children from low SES families [58-60], and there is evidence that low SES children are more likely to have poorer diets [77-79]. We observed that children in the high SES intervention group were much more likely to eat breakfast than those the low SES group. Breakfast is advocated as an important element of a healthy lifestyle for young people that is associated with reduced body weight and other positive health outcomes [44,80]. The limited evidence investigating the influence of SES on the effectiveness of school-based interventions to promote healthy weight is equivocal, possibly because studies have employed different measures of SES [81], which may be independently associated with body size outcomes [82]. By focusing on the promotion of healthy weight rather than weight loss per se, a favourable response was observed in the OW group. De-emphasising body weight but reinforcing and promoting healthy lifestyle behaviours related to energy balance may encourage more sustained changes in behaviour which can facilitate positive changes in body size [83].

This study demonstrated positive effects on body size outcomes and has several strengths. Over 75% of the study population consented to participate which reduced the risk of sampling bias. Randomisation occurred at the school level so as to reduce the risk of contamination to Comparison group children, and this cluster-randomised design was accounted for in the analyses. The intervention content was relevant to the local context of the schools and unlike other similar studies, was informed by the participants’ opinions and beliefs [22,23]. Few interventions of this nature have involved parents to reinforce their children’s engagement in healthy behaviours. Through regular family-focused homework tasks the children and their parents were provided with opportunities to learn together, thus messages about PA and healthy eating were promoted beyond the school environment and into the wider family unit. Furthermore, integration of the intervention with the existing curriculum and delivery by class teachers was a sustainable approach, that was undertaken at minimal financial cost. The low cost and simplicity of the intervention would make it easy to adopt and implement in others schools elsewhere in the UK.

The lack of an objective measure of food intake was a limitation of the study. The previous day food intake survey did not give a picture of dietary behaviours over a typical week. Moreover, the inability of the survey to record macronutrients did not allow energy intake to be estimated, and as a result we were unable to investigate the intervention’s effect on energy balance. For these reasons we were unable to confidently explain the positive effects on body size. Although teachers in the Intervention schools received training in use of the curriculum resource and homework tasks, there was no on-going record of lesson delivery or evaluation. Teachers provided feedback at the end of the study, but any inconsistencies in lesson delivery that occurred during the 20 week intervention period could not be addressed at the time, which increased the risk of intervention infidelity. Although the intervention training and curriculum resources were applicable to all Year 6 primary school teachers it is acknowledged that schools and individual teachers may have approached teaching the lessons in different ways, and this could have influenced the study results. Furthermore, although Comparison schools did not teach a specific unit of PSHE focused on healthy eating and PA, concepts related to these areas may have been touched on informally during other lessons such as science, food technology, and physical education. We do not believe that this would have impacted in a meaningful way on the eating and PA behaviours of the Comparison group, but acknowledge that this was not controlled. These points therefore should be taken into account when considering the generalisability of the findings. The higher levels of PA observed among the Intervention children at baseline suggest that the study design was a limitation, whereby the schools were allocated to Intervention or Comparison conditions prior to baseline data collection. An alternative approach is to randomise schools to conditions following initial comparisons of baseline data, but this was not possible due to the need to schedule Intervention teacher training combined with the number of weeks required for the intervention in relation to the available weeks across the school year. Furthermore, human resource constraints prohibited blinding of the research team to allocation of schools to the Intervention and Comparison conditions, and subsequent data analysis. Unlike some school-based interventions this study included a follow-up phase after the intervention lessons had ended. The duration of this though was limited to 10 weeks and therefore it could only be recognised as a short term period when intervention effects are likely to be stronger. To impact health, behaviour change needs to be sustained in the medium term (i.e., 6 months) and long term (i.e., 12 months and beyond). This pragmatic evaluation assessed the effectiveness of the CHANGE! intervention under ‘real-life’ conditions, and thus the design and limitations of the study reflected this.

## Conclusions

The CHANGE! school-based curriculum intervention resulted in significant effects on waist circumference, BMI z-scores, and LPA. CHANGE! was most effective among girls, overweight/obese, and high SES participants. The study findings add further support for the effectiveness of combined school-based physical activity and nutrition interventions. Effectiveness may be enhanced when such curriculum-based interventions include a formative phase to inform intervention design, involve parents in the children’s learning, focus on the positive aspects of PA and healthy eating rather than body weight or obesity prevention, have low participant burden, and are low cost. Further work is required to test intervention fidelity and the sustained effectiveness of this approach in the medium and long term.

## Competing interests

The authors declare that they have no competing interests.

## Authors’ contributions

SJF and LMB conceived and designed the study, assisted with data collection, undertook the analysis, and wrote the manuscript. IGD and AFH designed the study and commented on drafts of the manuscript. KAM, RG, and GW undertook data collection and commented on drafts of the manuscript.GS commented on drafts of the manuscript. EvS advised on the analyses and commented on drafts of the manuscript. All authors read and approved the final manuscript.

## Pre-publication history

The pre-publication history for this paper can be accessed here:

http://www.biomedcentral.com/1471-2458/13/626/prepub
